# Does a Supplemental Low-Protein Diet Decrease Mortality and Adverse Events After Commencing Dialysis? A Nationwide Cohort Study

**DOI:** 10.3390/nu10081035

**Published:** 2018-08-08

**Authors:** Chieh-Li Yen, Kun-Hua Tu, Ming-Shyan Lin, Su-Wei Chang, Pei-Chun Fan, Ching-Chung Hsiao, Chao-Yu Chen, Hsiang-Hao Hsu, Ya-Chun Tian, Chih-Hsiang Chang

**Affiliations:** 1 Kidney Research Center, Department of Nephrology, Chang Gung Memorial Hospital, Linkou Branch, Taoyuan 33305, Taiwan; b9102087@yahoo.com.tw (C.-L.Y.); 8902086@cgmh.org.tw (K.-H.T.); franwis1023@gmail.com (P.-C.F.); colinhua0123@gmail.com (C.-C.H.); Chaoyuclaire@gmail.com (C.-Y.C.); hsianghao@gmail.com (H.-H.H.); dryctian@yahoo.com (Y.-C.T.); 2 Department of Cardiology, Chang Gung Memorial Hospital, Chiayi branch, Chiayi 61363, Taiwan; mingshyan@cgmh.org.tw; 3 Clinical Informatics and Medical Statistics Research Center, College of Medicine, Chang Gung University, Taoyuan 33341, Taiwan; shwchang@mail.cgu.edu.tw; 4 Division of Allergy, Asthma, and Rheumatology, Department of Pediatrics, Chang Gung Memorial Hospital, Taoyuan 33341, Taiwan

**Keywords:** chronic kidney disease, ketoacids, low-protein diet, survival, nutrition, adverse events

## Abstract

Background: A beneficial effect of a ketoanalogue-supplemented low-protein diet (sLPD) in postponing dialysis has been demonstrated in numerous previous studies. However, evidence regarding its effect on long-term survival is limited. Our study assessed the long-term outcomes of patients on an sLPD after commencing dialysis. Methods: This retrospective study examined patients with new-onset end-stage renal disease with permanent dialysis between 2001 and 2013, extracted from Taiwan’s National Health Insurance Research Database. Patients who received more than 3 months of sLPD treatment in the year preceding the start of dialysis were extracted. The outcomes studied were all-cause mortality, infection rate, and major cardiac and cerebrovascular events (MACCEs). Results: After propensity score matching, the sLPD group (*n* = 2607) showed a lower risk of all-cause mortality (23.1% vs. 27.6%, hazard ratio (HR) 0.77, 95% confidence interval (CI) 0.70–0.84), MACCEs (19.2% vs. 21.5%, HR 0.86, 95% CI 0.78–0.94), and infection-related death (9.9% vs. 12.5%, HR 0.76, 95% CI 0.67–0.87) than the non-sLPD group did. Conclusion: We found that sLPD treatment might be safe without long-term negative consequences after dialysis treatment.

## 1. Introduction

Patients with chronic kidney disease (CKD) experience altered physical conditions in many aspects, including proteinenergy wasting (PEW) [1], mineral bone disorder [2,3], chronic inflammation [4,5], and uremic toxin accumulation [6,7]. Previous studies have demonstrated that the metabolism of protein is involved in most of these adverse changes [8,9], especially the uremic toxin accumulation. Thus, a diet therapy with restricted protein intake has been investigated as a way to retard the progression of CKD [10] and ameliorate uremia symptoms [11,12]. However, these studies have not yielded unequivocally beneficial results [13,14], and concerns of malnutrition have been raised regarding prolonged protein restriction without any nutritional supplementation in patients near dialysis, who already have a higher risk of PEW [1].

In recent decades, supplemented ketoanalogues of essential amino acids have been extensively used in patients on low-protein diets (sLPDs) (0.6–0.8 g/kg body weight per day) [15] or very low-protein diet (sVLPDs) (0.3–0.4 g/kg body weight per day) [16]. In vivo, via the transamination effect, ketoanalogues of essential amino acids can utilize circulating amino groups to transform themselves into essential amino acids to reduce possible malnutrition in those on a low-protein diet [17]. This process can consume circulation amino groups to prevent them from degrading into urea and other nitrogenous waste products. Because the transamination effect is most effective when restricted to essential amino acids [18], an sLPD or sVLPD theoretically can reduce metabolic waste products in patients with advanced CKD while also maintaining their proteinenergy status. After Walser, who first demonstrated the benefit of sVLPDs in retarding the progression of chronic kidney disease in 1975 [19], numerous randomized control trials have reported similar results in patients on an sLPD [20,21] or sVLPD [16]. Other studies [22,23] have also proved the benefit that dialysis is postponed for 1–2 years in addition to the retarding of CKD progression, which is believed to mainly result from the amelioration of uremic symptoms. Although many compelling results have been reported for sLPD and sVLPD in the retarding of renal function decline and postponing dialysis, one important aspect of this treatment still lacks analysis by large-scale, well-designed research: the effect on long-term survival. Furthermore, of the limited evidence so far on this issue comprises conflicting results. Most of the studies on sLPDs and sVLPDs have reported a stable or slightly improved nutritional status during the period of treatment [11,21,22]. However, some investigators have expressed doubt regarding whether this status can persist in consideration of the progression of malnutrition along with the start of dialysis [24,25]. A post hoc analysis of the Modification of Diet in Renal Disease (MDRD) study indeed demonstrated a slightly increased mortality rate in the sVLPD group after 10 years [26]. 

Because of the high prevalence of end-stage renal disease (ESRD) in Taiwan [27] and the country’s comprehensive nationwide database of medical record, we designed this study to use of the Taiwan National Health Insurance Research Database (NHIRD) to determine the long-term survival effect of low-protein diet supplemented with ketoanalogues in patients preceding dialysis. 

## 2. Materials and Methods

### 2.1. Data Source

The Taiwan National Health Insurance (NHI) program is a nationwide, single-payer, compulsory health care program covering approximately 99.9% of Taiwan’s population, which stood at approximately 23.37 million in 2014. The NHIRD contains the comprehensive health care information of insured patients, including disease diagnoses, outpatient visits, inpatient orders, drug prescriptions, procedure interventions, and registries of beneficiaries with specific conditions, but it does not include laboratory data. The disease diagnoses in the database are made according to the International Classification of Diseases, 9th Revision, Clinical Modification (ICD-9-CM). Data in the NHIRD that could identify specific patients or health care providers is scrambled before being released to researchers; thus, the current study, which was based on NHIRD data, had the need for consent waived by the Chang Gung Medical Foundation’s Institutional Review Board (approval number: 201800002B0). 

In the NHI program, patients with some specific chronic conditions, including ESRD, malignancies, and some autoimmune diseases, qualify for a catastrophic illness certificate, which relieves then of the need to make co-payments. To qualify for a certificate, a patient’s condition must be repeatedly verified by a peer review group based on pathologic findings, laboratory data, and clinical evidence. The Registry for Catastrophic Illness Patient Database (RCIPD) is an NHIRD subset that comprises the data of patients with a certificate, including those with ESRD and permanent dialysis.

### 2.2. Patient Selection and Study Design

**Figure 1:**
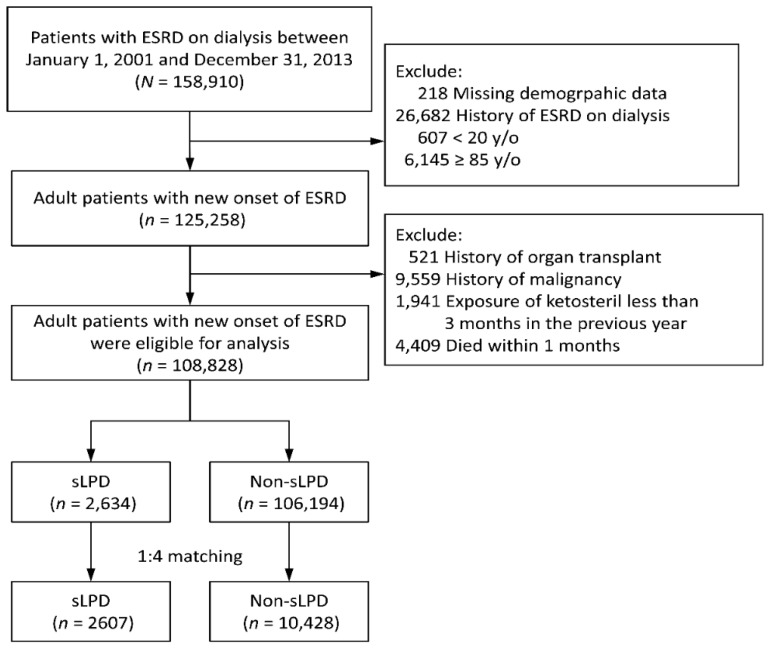
Inclusion of study patients. sLPD: ketoanalogue-supplemented low-protein diet. ESRD: end-stage renal disease.

To verify the relationship between an sLPD and long-term survival effect in permanent dialysis patients, we designed a population-based, nationwide, retrospective study. As shown in Figure 1, patients aged between 20 and 85 years with new-onset ESRD and who needed renal replacement therapy between 2001 and 2013 were identified from the RCIPD. 

The first date of hemodialysis or peritoneal dialysis was defined as the index date. Patients who died within 1 month of the index date or had a history of organ transplantation or malignancy were excluded. The extracted patients with new-onset dialysis were divided into two groups depending on whether more than 3 consecutive months of ketoanalogue supplementation was prescribed to them within the year before the index date. According to the NHI reimbursement regulations, low-dose ketoanalogue supplementation (a maximum daily dose of 6 tablets of ketosteril) can be prescribed without copayment in advanced CKD patients whose serum creatinine exceeds 6 mg/dL for more than 3 consecutive months, and this treatment should be combined with a low-protein diet (LPD) and evaluated by dietitians. The use and doses of ketosteril are determined by attending physicians according to the clinical situation, compliance, and nutritional status of each patient. If poor compliance with a low-protein diet or a 5% decrease of body weight occurs, the keoanalogues supplementation should be halted immediately. As a result of these regulations, exposure to ketosteril is a reasonable surrogate to represent a low-protein diet supplemented with ketoanaglogues. However, because distinguishing between poor compliance and poor nutritional status is difficult when determining the reason for early discontinuation, patients whose ketoanalogue supplementation persisted for less than three consecutive months were excluded from this study. 

### 2.3. Covariates and Study Outcomes

Diseases were detected using ICD-9-CM diagnostic codes. The covariates were age, sex, comorbidities, Charlson Comorbidity Index, hospitalization history, initial access to dialysis, initial dialysis type, and medications. Comorbidities were identified when reported for more than 2 outpatient visits or one inpatient stay within the previous year. The hospitalization histories were tracked to 1997. Medications were identified by the filling of a prescription at least twice or refilling a prescription for a chronic illness at least once in the previous 3 months. Most diagnostic codes used for these comorbidities have been validated in previous NHIRD-based studies [28,29].

The outcomes of primary interest were all-cause mortality and major cardiac and cerebrovascular events (MACCE), comprising acute myocardial infarction, acute ischemic stroke, intracerebral hemorrhage, heart failure, and cardiovascular death. Components of MACCE was detected based on the principal diagnosis of an emergency visit or hospitalization, most of these diagnostic codes for which have been previously validated [30,31,32]. All-cause mortality was defined by withdrawal from the NHI program. The secondary outcomes were infection/sepsis-related hospitalization and death. These outcomes were detected based on the principal or secondary diagnosis of an emergency visit or hospitalization. The codes of infection/sepsis have been reported in previous NHIRD-based studies [33,34]. 

### 2.4. Statistical Analysis

To achieve comparability between the study groups (sLPD vs. non-sLPD), we performed propensity score matching, in which each patient in the sLPD group was matched with four counterparts in the non-sLPD group. The propensity score was the predicted probability to be in the sLPD group derived from logistic regression, with the covariates being demographics, comorbidities, initial dialysis type, medications, and index date of ESRD (variables listed in Table 1). We adopted a greedy nearest neighbor algorithm with a caliper of 0.2 without replacement [35]. The quality of matching was verified using the absolute standardized mean difference (ASMD) between the groups after matching, in which a value less than 0.1 is considered to indicate a negligible difference between groups [36]. 

We compared the risk of all-cause mortality between groups using a Cox proportional hazard model. The risk of other time to event outcomes (i.e., infection death) between groups was compared using a subdistribution hazard model [37] that considered death during follow-up as a competing risk. Matching pairs were stratified [38] in both the Cox and subdistribution hazard models to consider the correlation among patients within the same matching pair. We plotted the cumulative incidence rate using subdistribution cumulative incidence function for time to event outcomes, except for all-cause mortality. We plotted Kaplan–Meier survival curves for all-cause mortality. A 2-tailed *p*-value of <0.05 was considered statistically significant and no adjustment of multiple testing (multiplicity) was conducted. All statistical analyses were performed using SAS (Statistical Analysis System) version 9.4 (SAS Institute, Cary, NC, USA), including ‘*psmatch*’ for propensity score matching and ‘*phreg*’ for survival analysis as well as the macro of ‘*%cif*’ for cumulative incidence function.

## 3. Results

### 3.1. Patient Characteristics

The main characteristics of the studied patients are provided in Table 1. A total of 108,828 adult patients with new-onset ESRD between 2001 and 2013 were eligible. Of them, 2634 had been on a low-protein diet supplemented with ketoanalogues (sLPD group) for more than 3 months during the year before the start of renal replacement therapy (index date), whereas the other 106,194 patients had not (non-sLPD group). Before matching, the sLPD group exhibited the following characteristics: younger, lower prevalence of diabetes mellitus and dementia, a lower comorbidity score, less frequent hospitalization, more frequent peritoneal dialysis, and less common prescriptions for antiplatelets, insulin, and more common prescriptions of iron supplements, pentoxifylline, vitamin D therapy, sodium bicarbonate, and calcium supplements. After matching, all the values of ASMD were less than 0.1, indicating only negligible differences between the groups in these clinical characteristics (Table 1).

### 3.2. Follow-Up Outcomes

**Figure 2:**
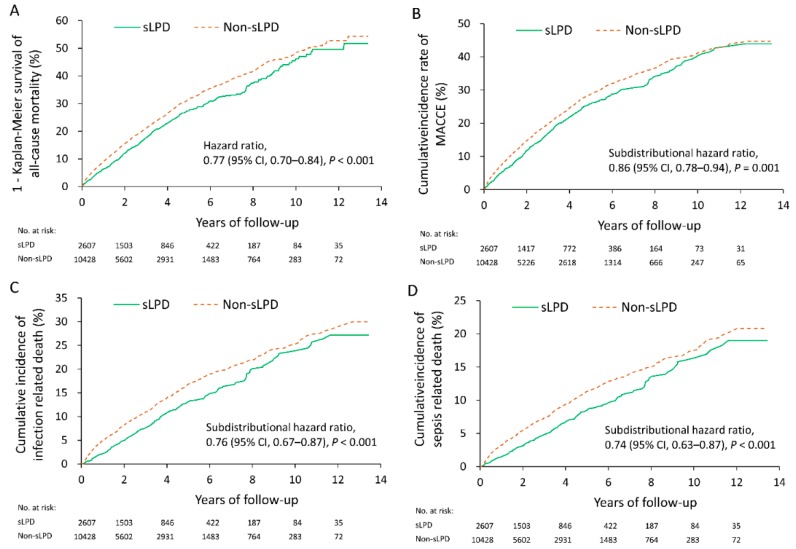
Kaplan–Meier survival curves for all-cause mortality (**A**) and cumulative incidence rates of major cardiac and cerebrovascular events (**B**), infection-related death (**C**), and sepsis-related death (**D**). CI: confidence interval.

After matching, 2607 patients remained in the sLPD group and 10,428 remained in the non-sLPD group. With a mean follow-up of 3.1 years (standard deviation 2.7 years), the sLPD group exhibited a lower rate of all-cause mortality compared with the non-sLPD group (23.1% vs. 27.6%, hazard ratio (HR) 0.77, 95% confidence interval (CI) 0.70–0.84). Regarding MACCEs, the sLPD group had a lower risk compared with the non-sLPD group (19.2% vs. 21.5%, HR 0.86, 95% CI 0.78–0.94). The Kaplan–Meier survival curves for all-cause mortality are presented in Figure 2A and the cumulative incidence rates of MACCEs are depicted in Figure 2B.

In respect to infection-associated adverse events, the sLPD group showed a significantly lower risk of infection-related hospitalization (38.7% vs. 43.0%, HR 0.83, 95% CI 0.78–0.89), infection-related death (9.9% vs. 12.5%, HR 0.76, 95% CI 0.67–0.87), sepsis-related hospitalization (15.9% vs. 21.0%, HR 0.71, 95% CI 0.64–0.79), and sepsis-related death (6.6% vs. 8.5%, HR 0.74, 95% CI 0.63–0.87). However, the risk of any kind of disability was not significantly different between the 2 groups (Table 2). Cumulative incidence rates of infection-related death and sepsis-related death are depicted in Figure 2C,D respectively.

### 3.3. Subgroup Analysis

**Figure 3:**
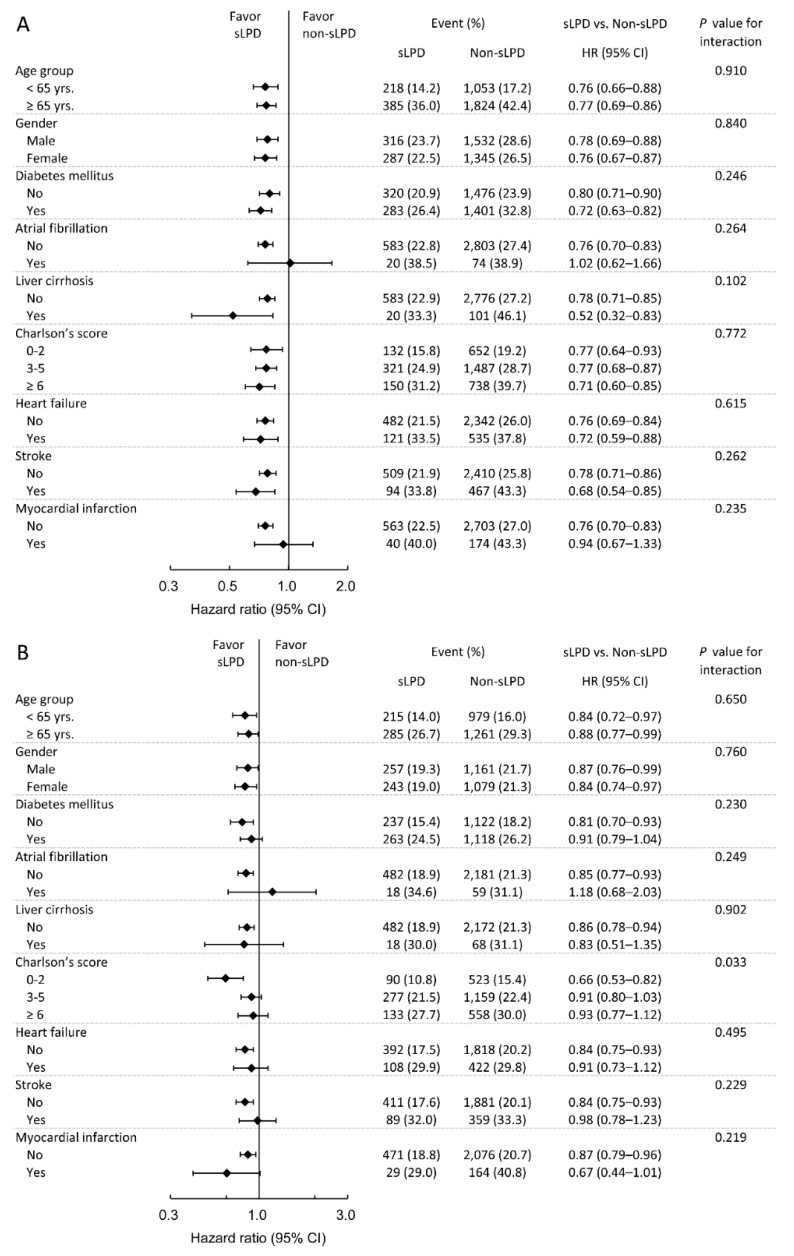
Prespecified subgroup analysis of all-cause mortality (**A**) and major cardiac and cerebrovascular events (**B**). HR: hazard ratio.

To further verify whether the protective effect of an sLPD was consistent among different clinical situations, we performed prespecified subgroup analyses for all-cause mortality, MACCEs, infection-related death, and sepsis-related death. Regarding all-cause mortality, the result showed that the benefit effect of sLPD on mortality was less apparent in patients with peritoneal dialysis (PD) (*p* for interaction = 0.013 the benefit of sLPD did not differ significantly across the levels of these subgroups (Figure 3A). To evaluate the impact of sLPD in patients received PD, we exam all PD patients before propensity score matching, the mortality rate was 18% (109/606) and 36.4% (4200/11547) in the sLPD and non-sLPD groups respectively, resulting in a hazard ratio of 0.67 (95% CI: 0.55–0.81) and adjusted hazard ratio of 0.71 (95% CI: 0.65–0.76) which adjusted for all covariates in Table 1. In respect to MACCEs, patients with higher comorbidity scores (≥3) exhibited less protective benefit of sLPD treatment (*p* for interaction = 0.033; Figure 3B). By contrast, patients with liver cirrhosis experienced a greater reduction in infection-related mortality compared with those without liver cirrhosis (*p* for interaction = 0.043; Figure S1A). Finally, regarding sepsis-related mortality, no significant differences in the subgroup analyses were observed (Figure S1B).

## 4. Discussion

Much evidence has been reported showing that LPDs and vLPDs supplemented with ketoanalogues can help retard CKD progression [16,20] and postpone dialysis [23,39], including one study [40] that utilized the same database as the current research. However, the evidence of a possible long-term survival benefit of this diet therapy is limited and conflicting. One secondary analysis [26] based on the patients of the MDRD study found an increment in mortality in patients with sVLPD, though some limitations may have resulted in flawed conclusions, including the long period without clinical follow-up between the end of the MDRD study secondary analysis as well as the imbalanced patient assignment in the original study. Recently, Vincenzo Bellizzi et al., [41] reported that an sVLPD during CKD was not associated with increased long-term mortality and may even reduce the risk in patients who are less than 70 years old or have no cardiovascular disease. That study also had some notable limitations, including a relatively small number (*n* = 184) of patients on sVLPD treatment in a tertiary nephrology clinic. Thus, we performed the current case–control cohort study based on the nationwide NHIRD to provide more information about the long-term survival effect of diet therapy.

Because of the observational cohort design of this study, the characteristics of who preferred to receive or not to receive diet therapy was able to be analyzed. The patients with an sLPD had relatively fewer comorbidities, a higher monthly income, and preferred to receive treatments with the potential to retard the progression of CKD, such as pentoxifylline and sodium bicarbonate. Notably, the significantly higher prevalence of patients who chose peritoneal dialysis for renal replacement therapy compared with those in the non-sLPD group may imply a better performance and self-care ability of these patients with diet therapy. The result showed that the benefit of sLPD on mortality was less apparent in patients with peritoneal dialysis (*p* for interaction = 0.013) after propensity score matching. However, it would not be appropriate to suggest that PD is not suitable for patients with sLPD. The possible explanation was that the timing of exposure of sLPD was before the selection of peritoneal dialysis (PD)/hemodialysis (HD) which was not randomly chosen by these patients and might bias the result. Therefore, to evaluate sLPD in patients received PD, we examined the all PD patients in original cohort and shown the mortality of sLPD is significantly better than non-sLPD in PD group. Thus, we would still recommend sLPD in patients who were prone to receive PD. Previously, protein restrictions were considered a treatment in liver cirrhosis owing to their contribution to ammonia production and the development of hepato encephalopathy. However, those recommendations were mostly based on the observational studies. Nowadays, LPD was not routinely suggested in patients with cirrhosis by new evidence [42]. In addition, in liver disease, due to the altered amino acid metabolism that occurs, the body’s amino acid profile and the ratio of branched-chain amino acids (BCAA): aromatic amino acids (AAA) changes to a higher AAA and lower BCAA. Supplementation with BCAA has been recommended for patients with hepatic encephalopathy [43,44]. However, there is no evidence of sLPD in patients with both cirrhosis and CKD. We suppose this is the first report of this phenomenon, so the related information is limited, and further studies are required. Notably, however, less than 3% of the patients in our study received the sLPD (*n* = 2634 vs. *n* = 106,194), which might suggest that achieving protein restriction is difficult in routine clinical practice. This difference was even more prominent among patients with comorbidities, especially diabetes mellitus and heart disease.

After taking advantage of 1:4 propensity matching analysis, which used every possible confounder to our knowledge, we analyzed the long-term survival effect based on 2 matched populations with a nearly homogenous distribution of patients. Notably, our study revealed that receiving sLPD treatment within 1 year before commencing dialysis not only did not increase all-cause mortality but was followed by a 23% decrease in mortality after renal replacement therapy. This effect may be attributable to the decrease in MACCEs and infection-related death, which were the 2 major causes of mortality in ESRD patients. Our research constitutes the first large-scale study investigating the potential protective effect of sLPD treatment against long-term cardiovascular death. Many investigators have reported that an sLPD or sVLPD has the potential to reduce some risk factors for cardiovascular disease, including calcium/phosphate imbalance [11,22], hypertension [45], proteinuria [46,47], and lipid disorders [48] in patients with chronic kidney disease. The major cardiovascular adverse events in patients with dialysis is believed to be partially causes by a cumulative effect of these metabolic imbalances during CKD [49]; therefore, diet therapy could be reasonably believed to reduce long-term cardiovascular adverse events by ameliorating these metabolic imbalances. Moreover, oral suppliment of ketoanalogue (KA) has benefit on protein metabolism and decrease the uremic symptoms, independently from LPD or VLPD [50,51]. The declining of GFR during the sLPD was also lower than the period of LPD alone [52]. Thus, the KA effect in sLPD group was acknowledged. In addition, there was no difference protective effect between DM and non-DM group treated with sLPD, that was also shown in the study by Piccoli et al. [53]. Notably, comorbidities would attenuate the protective effect of sLPD on MACCEs in subgroup analysis. Patients with CKD have multiple risk factors of MACCEs. In addition to traditional risk factor such as age, hypertension, diabetes, dyslipidemia, smoking, multiple non-traditional risk factors also exert significant contribution, such as malnutrition, inflammation, oxidative stress, anemia and mineral bone disease [54]. As CKD stage progress, the risk of MACCEs are expected to be affected by more confounder factors, therefore, the effect of sLPD treatment might be marginalized. Similarly, Vincenzo Bellizzi et al., [41] revealed a greater reduction of mortality by sVLPD treatment in patients without a history of cardiovascular disease. In addition, our study found a 24% reduction in infection-related death in patients with sLPD treatment. For decades, infection- and sepsis-related hospitalization and death in patients on permanent dialysis were considered a landmark of severe PEW [55,56]. Furthermore, the PEW inducing infection and death may be a complication that should mostly be of concern regarding patients who are on an LPD and are transitioning from CKD to ESRD. Our study also provided evidence of potential protective effects of an sLPD. Combined, the protective effects for MACCEs and infection-related death yielded a reduction of 23% in all-cause mortality in patients who received sLPD treatment preceding dialysis. These results are similar to those of a previous study by Vincenzo Bellizzi [41]; however, unlike that research, we found that the protective effect of sLPD treatment persisted among older patients and those with a CV history. The different populations (patients limited in third-degree nephrology clinic versus those of a nationwide cohort) and varying definitions of “old age” (>70 years versus ≥65 years) between these 2 studies may have contributed to these differences. 

The current study had some limitations that warrant mentioning. First, some important information, including serum albumin, creatinine, lipid profile, proteinuria, blood pressure control, daily protein intake, and body mass index, was not available in the NHIRD. Second, because of the study design, the sLPD duration and the ketoanalgue supplemenation dosage were beyond the scope of our research. Future studies should be performed to analyze the effect of treatment duration and dosage. Third, despite propensity matching analysis considering most relevant confounders we could identify, the observational and retrospective nature of this study entails some residual inherent limitations. Fourth, this study was performed based on a national database of Taiwan; the results of our study may thus not completely apply to other populations in consideration of dietary and genetic differences. 

## 5. Conclusions

In conclusion, this study found that most patients with advanced CKD did not adopt diet therapy, perhaps discouraged by its possible complication, such as PEW. Although our study revealed that sLPD treatment has long-term safety and may even have some protective effects after commencing dialysis, namely reduced risk of MACCEs and infection-related mortality, further validation of well-designed prospective study is warrant. We hope these results can help persuade more patients with advanced CKD to receive sLPD treatment and obtain the possible benefits of retarded CKD progression and postponed dialysis.

## Figures and Tables

**Table 1:** Characteristics of the all study patients.

	Before Matching			After Matching		
Characteristic	sLPD (*n* = 2634)	Non-sLPD (*n* = 106,194)	ASMD	sLPD (*n* = 2607)	Non-sLPD (*n* = 10,428)	ASMD
Age	60.9 ± 12.9	62.1 ± 13.6	0.09	60.9 ± 12.9	61.1 ± 13.9	0.01
Age ≥ 65 years, *n* (%)	1074 (40.8)	49,145 (46.3)	0.11	1068 (41.0)	4304 (41.3)	0.01
Male gender, *n* (%)	1343 (51.0)	54,473 (51.3)	0.01	1331 (51.1)	5359 (51.4)	0.01
Comorbidity in the previous year, *n* (%)						
Hypertension	2304 (87.5)	93,064 (87.6)	0.00	2279 (87.4)	9196 (88.2)	0.02
Diabetes mellitus	1074 (40.8)	62,480 (58.8)	0.37	1073 (41.2)	4265 (40.9)	0.01
Dyslipidemia	653 (24.8)	28,077 (26.4)	0.04	648 (24.9)	2529 (24.3)	0.01
Atrial fibrillation	52 (2.0)	3249 (3.1)	0.07	52 (2.0)	190 (1.8)	0.01
Peripheral arterial disease	70 (2.7)	3871 (3.6)	0.05	70 (2.7)	292 (2.8)	0.01
Liver cirrhosis	60 (2.3)	4285 (4.0)	0.10	60 (2.3)	219 (2.1)	0.01
Dementia	58 (2.2)	3191 (3.0)	0.05	58 (2.2)	226 (2.2)	0.00
Charlson Comorbidity Index score	3.7 ± 1.8	4.6 ± 2.0	0.47	3.8 ± 1.8	3.7 ± 1.7	0.02
Hospitalization history, *n* (%)						
Heart failure	361 (13.7)	27,567 (26.0)	0.31	361 (13.8)	1415 (13.6)	0.01
Stroke	278 (10.6)	18,395 (17.3)	0.19	278 (10.7)	1079 (10.3)	0.01
Myocardial infarction	100 (3.8)	7378 (6.9)	0.14	100 (3.8)	402 (3.9)	0.01
Infection-related hospitalization	1230 (46.7)	64,470 (60.7)	0.28	1223 (46.9)	4971 (47.7)	0.02
Initial dialysis type, *n* (%)						
Hemodialysis	2028 (77.0)	94,647 (89.1)	0.33	2015 (77.3)	8115 (77.8)	0.01
Peritoneal dialysis	606 (23.0)	11,547 (10.9)	0.33	592 (22.7)	2313 (22.2)	0.01
Medication, *n* (%)						
Aspirin/Clopidogrel	573 (21.8)	29,267 (27.6)	0.13	570 (21.9)	2306 (22.1)	0.00
ACEI/ARB	1314 (49.9)	48,098 (45.3)	0.09	1300 (49.9)	5142 (49.3)	0.01
Other antihypertensive agents	2147 (81.5)	82,373 (77.6)	0.10	2122 (81.4)	8510 (81.6)	0.01
Loop diuretics	1341 (50.9)	58,310 (54.9)	0.08	1334 (51.2)	5415 (51.9)	0.01
K-sparing diuretics	40 (1.5)	2387 (2.2)	0.05	40 (1.5)	178 (1.7)	0.02
OHA	606 (23.0)	34,346 (32.3)	0.21	606 (23.2)	2441 (23.4)	0.00
Insulin	403 (15.3)	23,087 (21.7)	0.17	403 (15.5)	1592 (15.3)	0.01
PPI	377 (14.3)	16,756 (15.8)	0.04	374 (14.3)	1520 (14.6)	0.01
NSAID (including COX2)	297 (11.3)	15,193 (14.3)	0.09	297 (11.4)	1184 (11.4)	0.00
Statin	548 (20.8)	22,687 (21.4)	0.01	543 (20.8)	2253 (21.6)	0.02
Fibrate or Gemfibrozil	65 (2.5)	4352 (4.1)	0.09	65 (2.5)	252 (2.4)	0.01
Iron supplement	639 (24.3)	17,960 (16.9)	0.18	634 (24.3)	2584 (24.8)	0.01
Pentoxifylline	666 (25.3)	11,794 (11.1)	0.37	644 (24.7)	2533 (24.3)	0.01
Vitamin D therapy	485 (18.4)	10,066 (9.5)	0.26	472 (18.1)	1800 (17.3)	0.02
Sodium bicarbonate	640 (24.3)	7686 (7.2)	0.48	615 (23.6)	2296 (22.0)	0.04
Calcium supplementation	1058 (40.2)	36,447 (34.3)	0.12	1041 (39.9)	4122 (39.5)	0.01
Steroid	256 (9.7)	8463 (8.0)	0.06	253 (9.7)	1057 (10.1)	0.01
Follow-up (years)	3.3 ± 2.8	4.0 ± 3.3	0.24	3.3 ± 2.8	3.0 ± 2.7	0.09

sLPD: low-protein diet with ketoacids; ASMD: absolute standardized mean difference; ACEI: angiotensin converting enzyme inhibitor; ARB: angiotensin receptor blocker; OHA: oral hypoglycemic agent; PPI: proton pump inhibitor; NSAID: non-steroidal anti-inflammatory drug; COX2: cyclo-oxygenase-2 inhibitor; Continuous data were given as mean ± standard deviation.

**Table 2:** Follow-up outcome at the last follow up.

	Event No. (%)		sLPD vs Non-sLPD	
Outcome ^#^	sLPD (*n* = 2607)	Non-sLPD (*n* = 10,428)	HR (95% CI)	*p*-Value
All-cause mortality	603 (23.1)	2877 (27.6)	0.77 (0.70–0.84)	<0.001
Cardiovascular composite adverse event ^§^	500 (19.2)	2240 (21.5)	0.86 (0.78–0.94)	0.001
Acute myocardial infarction	87 (3.3)	327 (3.1)	1.05 (0.83–1.33)	0.695
Acute ischemic stroke	114 (4.4)	525 (5.0)	0.86 (0.70–1.05)	0.135
Intracerebral hemorrhage	34 (1.3)	181 (1.7)	0.74 (0.51–1.07)	0.107
Heart failure	90 (3.5)	418 (4.0)	0.85 (0.68–1.07)	0.156
Cardiovascular death	310 (11.9)	1366 (13.1)	0.88 (0.78–0.99)	0.039
Infection-related hospitalization	1009 (38.7)	4479 (43.0)	0.83 (0.78–0.89)	<0.001
Infection death	259 (9.9)	1308 (12.5)	0.76 (0.67–0.87)	<0.001
Sepsis-related hospitalization	415 (15.9)	2188 (21.0)	0.71 (0.64–0.79)	<0.001
Sepsis death	171 (6.6)	890 (8.5)	0.74 (0.63–0.87)	<0.001
Disability	788 (30.2)	3274 (31.4)	0.94 (0.87–1.01)	0.098
PRBC (admission)	1451 (55.7)	6004 (57.6)	0.91 (0.86–0.96)	0.001

sLPD: low-protein diet with ketoacids; HR: hazard ratio; CI: confidence interval; PRBC: packed red blood cells; ^§^ Anyone of acute myocardial infarction, acute ischemic stroke, intracerebral hemorrhage, heart failure and cardiovascular death; # Estimated using subdistribution hazard model which considered all-cause mortality as a competing risk.

## Supplementary Materials

The following are available online at http://www.mdpi.com/2072-6643/10/8/1035/s1, Figure S1: Prespecified subgroup analysis of infection-related death (A) and sepsis-related death (B).
