# Supplementary material for: Does a Supplemental Low-Protein Diet Decrease Mortality and Adverse Events After Commencing Dialysis? A Nationwide Cohort Study
Source: Nutrients. 2018 Aug 8;10(8):1035. doi: 10.3390/nu10081035 (PMC6115816; doi:10.3390/nu10081035)
Supplement: Supplementary file 1 [file nutrients-10-01035-s001.pdf]

nutrients

Supplementary Materials: Does a Supplemental Low-Protein Diet Decrease Mortality and Adverse Events After Commencing Dialysis? A Nationwide Cohort Study

Chieh-Li Yen [1,†], Kun-Hua Tu [1,†], Ming-Shyan Lin [2],Su-Wei Chang [3,4], Pei-Chun Fan[1], Ching-Chung Hsiao [1], Chao-Yu Chen[1], Hsiang-Hao Hsu [1], Ya-Chun Tian [1], Chih-Hsiang Chang [1,*]

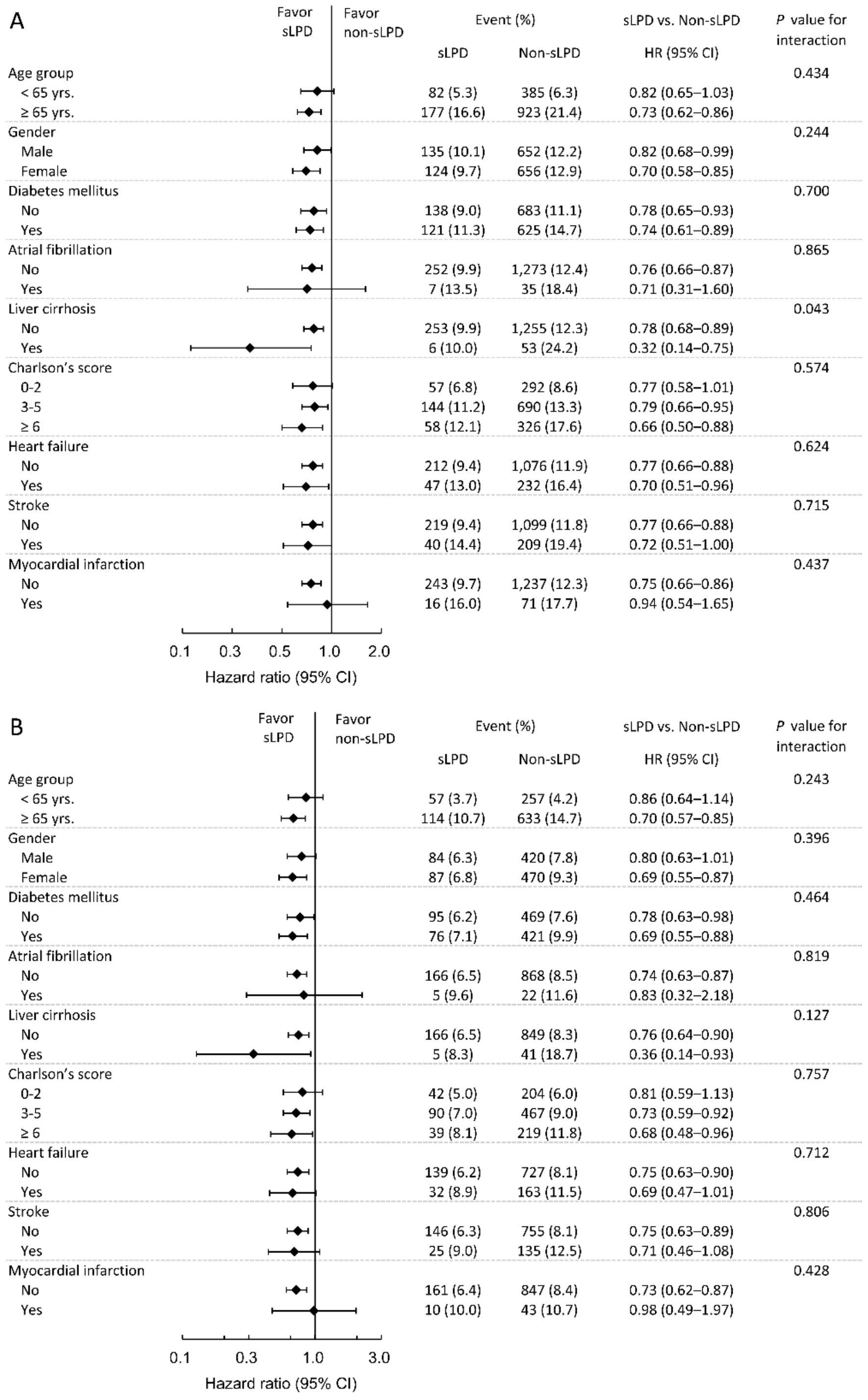

Figure S1. Presepcified subgroup analysis of infection-related death (**A**) and sepsis-related death (**B**).
